# A qRT-PCR assay for the expression of all *Mal d 1* isoallergen genes

**DOI:** 10.1186/1471-2229-13-51

**Published:** 2013-03-23

**Authors:** Giulia Pagliarani, Roberta Paris, Paul Arens, Stefano Tartarini, Giampaolo Ricci, Marinus MJ Smulders, W Eric van de Weg

**Affiliations:** 1 Wageningen UR Plant Breeding, Plant Research International, Droevendaalsesteeg 1, Wageningen, PB, 6708, The Netherlands; 2 Department of Fruit Tree and Woody Plant Sciences, University of Bologna, Viale Fanin 46, Bologna, 40127, Italy; 3 Department of Paediatrics, University of Bologna, Via Massarenti 11, Bologna, 40138, Italy; 4 Present address: Consiglio per la Ricerca e la sperimentazione in Agricoltura-Centro di Ricerca per le Colture Industriali, via di Corticella 133, Bologna, 40128, Italy

**Keywords:** Apple allergy, OAS, Mal d 1, Bet v 1, PR-10, gene family, qRT-PCR

## Abstract

**Background:**

A considerable number of individuals suffer from oral allergy syndrome (OAS) to apple, resulting in the avoidance of apple consumption. Apple cultivars differ greatly in their allergenic properties, but knowledge of the causes for such differences is incomplete. Mal d 1 is considered the major apple allergen. For Mal d 1, a wide range of isoallergens and variants exist, and they are encoded by a large gene family. To identify the specific proteins/genes that are potentially involved in the allergy, we developed a PCR assay to monitor the expression of each individual *Mal d 1* gene. Gene-specific primer pairs were designed for the exploitation of sequence differences among *Mal d 1* genes. The specificity of these primers was validated using both *in silico* and *in vitro* techniques. Subsequently, this assay was applied to the peel and flesh of fruits from the two cultivars ‘Florina’ and ‘Gala’.

**Results:**

We successfully developed gene-specific primer pairs for each of the 31 *Mal d 1* genes and incorporated them into a qRT-PCR assay. The results from the application of the assay showed that 11 genes were not expressed in fruit. In addition, differential expression was observed among the *Mal d 1* genes that were expressed in the fruit. Moreover, the expression levels were tissue and cultivar dependent.

**Conclusion:**

The assay developed in this study facilitated the first characterisation of the expression levels of all known *Mal d 1* genes in a gene-specific manner. Using this assay on different fruit tissues and cultivars, we obtained knowledge concerning gene relevance in allergenicity. This study provides new perspectives for research on both plant breeding and immunotherapy.

## Background

Apple allergy is an issue for a growing number of European citizens. As one of the most prevalent food allergies, apples rank fourth out of 24 foods examined in an extensive Pan-European survey and first among *Rosaceae* fruits [1]. Thus, although the apple is generally a healthy component in the human diet, an increasing number of individuals cannot eat this fruit. The allergenic properties of apple cultivars differ greatly [2,3], but knowledge of the genetic basis for low and high allergenicity remains incomplete.

Of the 4 classes of allergens currently identified in apple, Mal d 1 is thought to be the major allergen in Central and Northern Europe [4,5]. At the genetic level, *Mal d 1* is a complex gene family composed of 31 different loci, each of which codes for a different isoallergen [6]. Moreover, for each isoallergen gene, there are a series of slightly different alleles that might encode for isoallergen variants, which increases the variability in Mal d 1 proteins [6-8]. Accumulating evidence has shown that isoallergens might differ greatly in their allergenic properties, but it is still unclear which of these proteins are more involved in allergy. Several approaches have been used to quantify Mal d 1 content or gene expression; however, none of these studies covered the full set of Mal d 1 isoallergens or *Mal d 1* genes. Mal d 1, similarly to Bet v 1, is unstable to pepsin digestion, and IgE reactivity to Mal d 1 proteins is absent following the heat treatment of fruits [9]. The sensibility of Mal d 1 to high temperature and proteases hinders its proteomic analysis. Moreover, the food matrix and contaminants might affect the protein extraction. Until now, proteomics have been primarily used to quantify the total amounts of Mal d 1 content in apple fruit, without distinguishing isoallergens or variants or making distinctions within an incomplete pool of isoallergens [10-14]. Currently, there are only a few recombinant allergens derived from fruits and vegetables that are commercially available for immunological detection [15], with variable antibody specificities [16].

PCR-based expression studies are not subject to these limitations, and in particular, quantitative Real-Time PCR (qRT-PCR) is a fast, highly sensitive and reproducible technique to study gene expression. Previous studies of some *Mal d 1* genes revealed the tissue- and cultivar-specific expression of *Mal d 1* genes [17-19], and differential effects of environmental conditions [20,21] on the transcription of these genes have been reported. However, these studies have not covered the entire gene family, nor have they sufficiently demonstrated the gene specificity of the PCR primer pairs used.

Thus, there is a need to characterise the role of each individual isoallergen to understand the apple allergy mechanism. Therefore, the aim of this study was to examine the expression of these genes by implementing the qRT-PCR approach. A comprehensive, robust, sensitive and affordable assay for studying the expression of all 31 known *Mal d 1* genes individually was developed. We successfully used this assay to generate the complete expression profile of all *Mal d 1* isoallergen genes in the fruits of two cultivars.

## Results

### Alignment of *Mal d 1* genes

**Figure 1:**
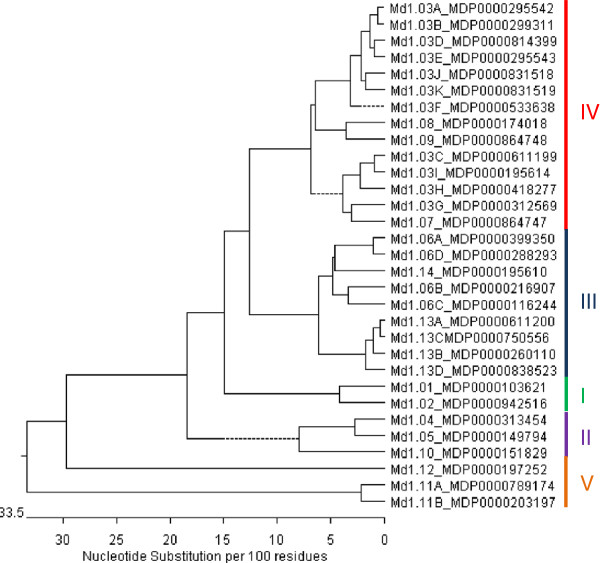
***Mal d 1 *phenetic tree.** Neighbour-joining tree based on the coding sequence of the 31 *Mal d 1* isoallergen genes retrieved from the ‘Golden Delicious’ genome sequence (Additional file 2). The sequences are presented using the names of the related genes and the accession numbers obtained from Apple GBrowse - Malus x domestica v1.0 [22]. The Roman numerals (I-V) and colored lines identify the subfamilies.

A total of 380 *Mal d 1* DNA and EST sequences were retrieved from the literature and databases (Additional file 1) covering all 31 known *Mal d 1* genes described in [6]. Many sequences obtained from different apple cultivars were already annotated as *Mal d 1* alleles. For the others, according to the level of similarity, it was possible to identify new alleles of known genes. Subsequent to alignment, the level of similarity among the coding sequences (cds) of different genes ranged from 53.1% to 97.7% and from 95% to 99.8% for the different alleles within a gene. At the protein level, the sequence identity between isoallergens ranged from 37% (Mal d 1.08 and Mal d 1.11A, 102 AA substitutions) to 96% (Mal d 1.06A and Mal d 1.06D, 7 AA substitutions). The alignment of the 31 coding sequences retrieved from the ‘Golden Delicious’ whole genome sequence (Additional file 2) was used to generate a phenetic tree (Figure 1). This tree showed 5 clades, 4 of which have been previously described and characterised as subfamilies I-IV [7] and one clade, subfamily V, that has been classified for the first time in the present study. The three genes in subfamily V (*Mal d 1.11A*, *Mal d 1.11B* and *Mal d 1.12*) are the most distant within the *Mal d 1* family.

### Primer design

**Figure 2:**
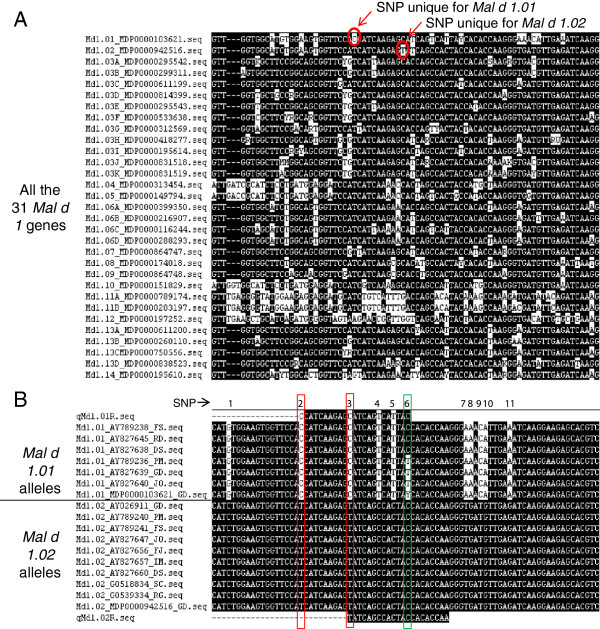
**Primers design strategy. A**) Part of the coding sequence alignment of the 31 *Mal d 1* isoallergen genes was retrieved from the ‘Golden Delicious’ genome sequence (Additional file 2). The sequences were named according to their related *Mal d 1* genes and the accession numbers retrieved from the Apple GBrowse - Malus x domestica v1.0 [22]. The white marked nucleotides highlight the mismatched residues in the consensus sequence. The SNPs specific for the loci *Mal d 1.01* and -*1.02* are indicated with red circles. **B**) Part of the alignment on all allelic gDNA sequences for *Mal d 1.01* and -*1.02* was obtained from the literature [8]. The sequences of the two reverse primers for *Mal d 1 .01* and -*1.02* are also included in the alignment. The sequences were named according to their related *Mal d 1* genes, ID numbers from the database and genotypic origins: FS: Fiesta; RD: Red Delicious; DS: Discovery; PM: Prima; GD: Golden Delicious; JO: Jonagold; FJ: Fuji; IM: Ingrid Marie; SC: Suncrisp; RG: Royal Gala. The SNP positions are indicated with successive numbers from 1 to 11. The red boxes indicate the two locus-differentiating SNPs exploited for primer design; the green box highlights an allele-differentiating SNP.

The development of this assay began with the design of specific primer pairs. The alignment of the 31 *Mal d 1* sequences from ‘Golden Delicious’ (Figure 2A) highlights SNPs specific for only one gene. The robustness of these SNPs across alleles of the same gene was examined among the other allelic sequences in the alignment, using the ‘Golden Delicious’ sequences as a framework. An average of four gene-differentiating SNPs per sequence was detected and only these SNPs were exploited for primer design. For example, the reverse primer for *Mal d 1.02* is located in a region in which a SNP differentiates the *Mal d 1.02* gene from all other genes (Figure 2A). The alignment of all allelic sequences for *Mal d 1.02* (Figure 2B) shows that no additional allele-differentiating SNPs were present in this region; thus, this region was an excellent candidate for primer development. Figure 2B shows an example of the reverse primer for *Mal d 1.01*. In addition to the gene-differentiating SNP (SNP nr. 2) targeted for primer design, this primer contained one allele-differentiating SNP (SNP nr. 6) at the 5’ end. In general, regions containing only gene-differentiating SNPs were preferred for primer design; however, these regions were not always available. Thus, 12 of the 31 primer pairs contained allele-differentiating SNPs (Table 1). To ensure that these SNPs would not affect the PCR amplification, the allele-differentiating SNPs were accepted only if positioned at the 5’ end of only one of the two primers. The procedure for the primer selection was performed for each of the 31 *Mal d 1* genes (Table 1).

**Table 1:** ***Mal d 1 *specific primer pairs for qRT-PCR analysis**

**Gene**	**Primer name**	**Sequence 5′-3′**	**SNP cons**^**a**^	**SNP gene**^**b**^	**SNP allele**^**c**^	**Start position**	**Length (bp)**	**Primer conc.**	**Ta**	**Tm**	**Slope**
***Mal d 1.01***	qMd1.01/02F	GATTGAAGGAGATGCT**T**T**GA**C**A**	5	-	-	258	103	100	63	80.5	−0.079
	qMd1.01R	GTA**A**TG**A**CTG**A**TGCTCTTGATG***G***	-	1	1						
***Mal d 1.02***	qMd1.01/02F	GATTGAAGGAGATGCT**T**T**GA**C**A**	4	-	-	258	111	100	62	81.0	−0.206
	qMd1.02R	TTGGTGTGGTAGTGGCTG**A**T***A***	1	1	-						
***Mal d 1.03A***	qMd1.03AF	A**T**C***T***GAGTTCACCTCCGTCAT***T***	1	2	-	21	96	70	63	81.0	−0.057
	qMd1.03AR	ACTGCTTGTGGTGGAATCTT***T***	-	1	1						
***Mal d 1.03B***	qMd1.03BF	TGTTTTCACATACGAA**T**CCGA***A***	1	1	-	6	167	100	63	83.5	−0.570
	qMd1.03BR	TGATCTTCTT**A**ATGGTTCCTAC***G***C	1	1	1						
***Mal d 1.03C***	qMd1.03CF	CTCCGA**A**ACAATTGAGAA**A**ATCT**G**	3	-	1	276	79	100	63	80.5	−0.120
	qMd1.03CR	GCTGGTGCTCTTGATGA**T**G***C***	1	1	-						
***Mal d 1.03D***	qMd1.03D/EF	ATACGAATCCGAGTTCACCTC**T**	1	-	-	15	156	70	62	83.0	0.005
	qMd1.03DR	ATCTTCT**T**AATGGTTCCAACTCC***T***	1	1	-						
***Mal d 1.03E***	qMd1.03D/EF	ATACGAATCCGAGTTCACCTC**T**	1	-	2	15	169	70	62	83.0	−0.116
	qMd1.03ER	TTCACCGAAGTTGATCTTCTT**A**AT***A***	1	1	-						
***Mal d 1.03F***	qMd1.03FF	CACAG**A**ATTGA***C***GGG**G**T***G***	2	2	-	208	119	70	63	81.0	−0.122
	qMd1.03FR	CCGGAAGC***G***ACCAAC**T**T**A**	2	1	1						
***Mal d 1.03G***	qMd1.03GF	AT**T**ATCAAGAGCACCAG***T***CA**C**TAC***T***	2	2	-	337	122	70	62	81.0	−0.254
	qMd1.03GR	TCCAAGAGGTAGTTCTCAATCA**A**	1	-	-						
***Mal d 1.03H***	qMd1.03HF	AA**A**ATCT**G*C***TA**C**GAGACTAAGTTG***A***	3	2	-	277	173	100	61	83.5	−0.431
	qMd1.03HR	**T**GGTGCTCCAAGAGGTAGTT***T***	1	1	-						
***Mal d 1.03I***	qMd1.03IF	CCCCAAGATTGCACCACA***T***	-	1	-	93	228	100	61	81.5	−0.230
	qMd1.03IR	GCCACCAACTTAGTCTC**G**TAA**C**A***A***	2	1	-						
***Mal d 1.03J***	qMd1.03JF	GCA**T**CA**C**CCACTACCACAC***A***	2	1	-	347	134	70	61	82.0	−0.105
	qMd1.03JR	CGAGCTGTAGG***A*G**TC***T*T**G**G**T***T***	3	3	-						
***Mal d 1.03K***	qMd1.03KF	CA**T**CA**G**CCACTACCACAC***A***AA	2	1	-	348	128	100	61	81.5	−0.431
	qMd1.03KR	TGTATGCATC**CT**GGTGCTC***T***	2	1	1						
***Mal d 1.04***	qMd1.04F	**G**G**G**TATGT**T**AAGCA***A***AGG**G**T**C*A***	5	2	-	196	193	100	61	80.5	−0.103
	qMd1.04R	TGATCTCAACATCACCCTT**A**G***C***	1	1	-						
***Mal d 1.05***	qMd1.05F	ATCAA**A**C**C**ACTAG**T**CACT***G***CCA**T**	4	1	1	343	124	70	63	82.5	−0.141
	qMd1.05R	GGT**TGG**CCA**CA**AGGTAG**G**T***T***	6	1	-						
***Mal d 1.06A***	qMd1.06AF	CTA**T**A**GC**TA**T**AG**C**TTGATTGAAGG***G***	5	1	-	243	167	100	61	80.5	−0.203
	qMd1.06AR	T**T**CCAACCTTAACATG**T**TCTTC**T**	3	-	1						
***Mal d 1.06B***	qMd1.06BF	AAACCGA**A**T**A**C**G**C***A***TCC***A***T***T***	3	3	1	20	106	100	61	81.5	−0.012
	qMd1.06BR	**A**C**AG**T**T**TT**G**ACTGCTTGTGG**A**G	6	-	-						
***Mal d 1.06C***	qMald1.06CF	GC**T**CCACAAGCAGT**C**AA**A**A**CT**	5	-	-	103	116	70	63	80.5	−0.250
	qMald1.06CR	TCAA**C**C**T**TGTGCTTCACATA***A***C**T*A***	3	2	-						
***Mal d 1.06D***	qMd1.06DF	CCC**T**CCTGCTAGGTTGT**A*TT***	2	2	-	42	100	70	61	80.5	−0.005
	qMd1.06DR	TCC**C**TC**G**AG**A**ATTTCA**A**C**AG**	6	-	-						
***Mal d 1.07***	qMd1.07F	CAACTTTGT**G**TAC***C***AGTACAGT**G**T***C***	2	2	-	234	126	100	61	81.5	−0.201
	qMd1.07R	TAGTGGCTG**A**TGCTCTTGAT***A***A**C**	2	1	-						
***Mal d 1.08***	qMd1.08F	***T***CTTCGGTGAAGGTAGCACA***A***	-	2	-	173	200	100	61	81.5	−0.390
	qMd1.08R	ACCCTT**A**GTGTGGTAGTGGC***AT***	1	2	-						
***Mal d 1.09***	qMd1.09F	TTTTCACATACGAATCCGAGT***C***	-	1	-	8	126	100	61	84.0	−0.265
	qMd1.09R	GGAT**C**TCA**A**CGCTCTTCAC***A***	2	1	-						
***Mal d 1.10.***	qMald1.10F	CAA**G**GC**T**TT***C*A**T**C**CA**C**GA***C***	5	2	1	60	157	100	61	83.0	−0.158
	qMald1.10R	**G**AT**T**CTGTGCTT***T***ACA***A***A**C**C**C*T***	4	3	-						
***Mal d 1.11A***	qMald1.11AF	**G**G**A**GG**A**T**G**CATC**TGTC**A**TTT*G***	11	1	-	343	130	100	62	79.5	−0.018
	qMald1.11AR	CCA**T**GAG***A***TAG**GCT**TC**C**A**A*A***A**CT**	8	2	-						
***Mal d 1.11B***	qMd1.11BF	CAGC**ACA**TAC**A**A**AG**CCAA**A**G**A*C***	8	1	-	363	125	100	61	81.0	−0.106
	qMd1.11BR	T**T**TA**T**GC**GCG**AGGGTG***T*G**	6	1	-						
***Mal d 1.12***	qMd1.12F	**GCT**TACA**C**TTTG***G***TTGAAGGAGA***AC***	4	3	-	247	171	100	62	76.0	−0.227
	qMd1.12R	**C*C***TGCCAGC***T***TT***T***A**TT**T**CT**TC***C***	5	4	-						
***Mal d 1.13A***	qMd1.13AF	GTGTTGGAACCATCAAGAA**GA**T***T***A***G***	2	2	-	149	124	100	61	78.0	−0.216
	qMd1.13AR	**A**CATCTCCTTCAATCAAACTGTA***A***T	1	1	-						
***Mal d 1.13B***	qMd1.13BF	CGAAGATAACTTTGTCTACA**AC**CA***T***	2	1	-	258	137	70	61	81.5	0.003
	qMd1.13BR	GCTCTTCCTTGATCTCAACATC**T*T***	1	1	-						
***Mal d 1.13C***	qMd1.13CF	GA**A**TTC**G**CCTC**A**GTC**TC**C***A***	5	1	-	25	186	70	61	82.0	0.087
	qMd1.13CR	GTGCTTCACATAGCTGTAT**TCA**CT***T***	3	1	-						
***Mal d 1.13D***	qMd1.13DF	TGTTGGAACCATCAAGAAGAT***A***A**GT**	2	1	-	150	124	100	61	78.5	−0.163
	qMd1.13DR	**GA**CATCTCCTTCAATCAAACTGTA***G***	2	1	-						
***Mal d 1.14***	qMd1.14F	GGTGAAGG**G**AG**TGA**ATACA**A**CTAT***A***	6	1	-	178	185	100	61	79.0	−0.470
	qMd1.14R	TGGTAATGGCTA**A**TG**T**TCTTGAT***AC***	2	2	2						

^a^SNPs to the consensus sequence showed in bold; ^b^gene-differentiating SNPs showed in italics and ^c^allele-differentiating SNPs showed by underlining. The last column contains the slopes of the curves obtained by plotting log input vs ΔCt (Ct *Mal d 1* gene - Ct *actin*). The values between −0.1 and 0.1 are underlined.

### Primer validation and qRT-PCR optimisation

**Figure 3:**
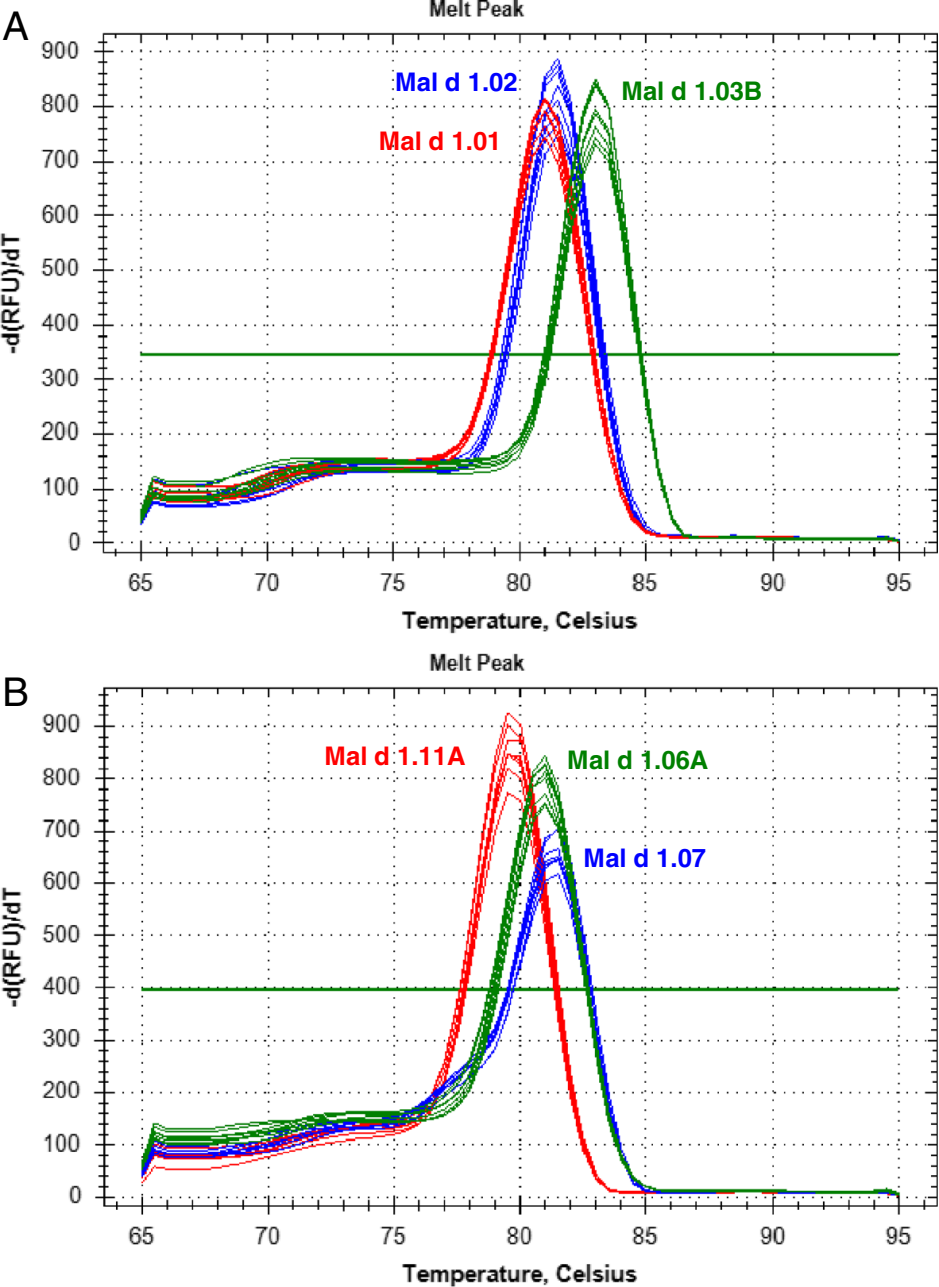
**Melting curve analyses of two different qRT-PCR reactions.** The negative first derivative of the change in fluorescence is plotted as a function of temperature. The single peak for each primer pair indicates the presence of only one PCR product. **A**) Melting curves for *Mal d 1.01* (red), *Mal d 1.02* (blue) and *Mal d 1.03B* (green) amplicons. **B**) Melting curves for *Mal d 1.11A* (red), *Mal d 1.07* (blue) and *Mal d 1.06A* (green) amplicons.

The gene-specificity of the designed primer pairs was validated in four ways. Firstly, an *in silico* validation through blasting the primer sequences to known reference sequences ensured that these primers perfectly matched only with sequences corresponding to the target genes (data not shown). Only the primers that generated perfect matches were assessed in the second validation step performed through end-point PCR on genomic DNA; only the primer pairs that produced a single clear band were maintained. Thirdly, the direct sequencing of the *Mal d 1* amplicons obtained from 10 apple cultivars was performed to ensure that only the specific target sequences were amplified (Additional file 3). Amplicons generated from gDNA were sequenced because of the higher level of complexity of gDNA compared to cDNA and this allowed to guarantee the primers specificity for all the *Mal d 1* members, independently from their expression. Only the primers that had a unique sequence corresponding to the target gene were assessed in the last step of validation. In some cases, this specificity was obtained after increasing the annealing temperature (Ta) within the range of 61-63°C and/or decreasing the final primer concentration from 100 nM to 70 nM. Some of the amplicon sequences revealed new *Mal d 1* alleles for the known genes (Additional file 3). Fourthly, the amplification specificity was validated through melting curves obtained with a single high peak (Figure 3), indicating the absence of nonspecific amplifications or primer dimers. The primer pairs that met this last criterion were selected for the qRT-PCR assay. The melting temperatures (Tm) for these primers are reported in Table 1.

Expectedly, significant variations in PCR efficiency were detected among the *Mal d 1* amplicons (Table 1, slopes in the last column) due to the forced positions of the primers to the targeted SNPs and variations in the PCR conditions to ensure specificity. However, the “Standard curve method” was chosen to neutralise these differences in efficiency. Particularly, this method has to be used when the slope values of the curves obtained by plotting log input vs dCt (Ct *Mal d 1* gene – Ct *actin*) are outside the −0.1 to 0.1 range [23], as in the case of this study (Table 1). The standard curves were of high quality, as a linear relationship between the input DNA and the Ct values across the standard samples (serial dilutions) was observed, with a squared regression coefficient close to 1 for all genes. Moreover, the use of standard samples in all the qRT-PCR reactions facilitated the evaluation of the reproducibility among experiments and the integration of the data.

**Figure 4:**
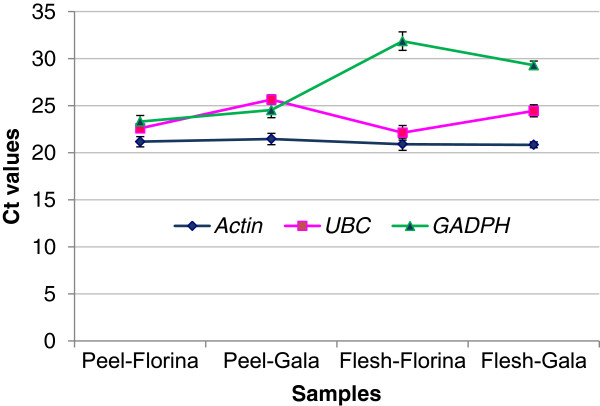
**Ct values of three putative reference genes in ‘Florina’ and ‘Gala’ peel and flesh.** The Ct values obtained after qRT-PCR amplification with the primers for *actin*, *UBC* and *GAPDH* in ‘Florina’ and ‘Gala’ samples are plotted in the chart. Each data point is the average of two biological replicates and the bars indicate the standard deviation.

Finally, the comparison of the gene expression in ‘Florina’ and ‘Gala’ of *actin*, *UBC* and *GAPDH* was performed in order to choose the most suitable reference gene for this study. *Actin* showed a highly stable expression among tissues and cultivars (Figure 4) and thus, it was an optimal reference gene for this study.

### Application: *Mal d 1* expression profiles in different apple tissues

In this study, we applied this new qRT-PCR assay to the peel and flesh of apple fruits from the cultivars ‘Florina’ and ‘Gala’. These two cultivars were selected because they differ in their allergenic characteristics in skin prick tests [24]. Moreover, both cultivars have been extensively used in the breeding of new apple cultivars and are subject to GM studies concerning the enhancement of ‘Gala’ resistance to the main disease in apple production, the fungus *Venturia inaequalis*, through gene transfer without raising *Mal d 1* levels [25].

**Figure 5:**
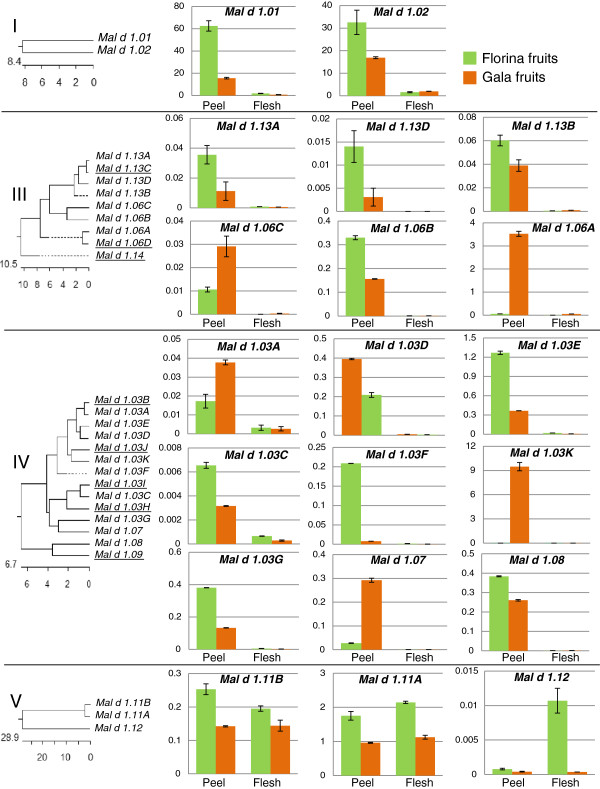
**Expression profiles of the *Mal d 1 *genes in the peel and flesh of apple fruits.** The genes were grouped into subfamilies and reported according to the order obtained in the phenetic tree. The branches of the phenetic trees for each subfamily (I-V) are reported on the left. The underlined genes were not expressed (charts not reported: subfamily II was not reported because none of its genes were expressed). The green bars indicate the results for ‘Florina’, and the orange bars indicate the results for ‘Gala’. The expression levels were normalised in respect to *actin* and reported in Arbitrary Units (A.U.). Each data point is the average of the final expression values of two biological replicates.

Among the 31 *Mal d 1* genes, 11 were not expressed in any fruit tissue. These genes included all of the genes of subfamily II and several genes of subfamilies III and IV (respectively *Mal d 1.06D*, -*1.13C* and -*1.14,* and *Mal d 1.03B*, -*1.03H,* -*1.03I*, -*1.03J*, and *-1.09*) (Figure 5). The 20 genes expressed in these fruits included all of the genes of subfamilies I and V and some of the genes of subfamilies III and IV (respectively *Mal d 1.06A,* -*1.06B,* -*1.06C,* -*1.13A,* -*1.13B,* -*1.13D* and -*1.03A,* -*1.03C,* -*1.03D,* -*1.03E,* -*1.03K,* -*1.03F,* -*1.03G,* -*1.07,* -*1.08*). A significant variation in the transcript level among genes, tissues and cultivars was observed (P<0.0001).

Concerning the variations in expression among the genes, the two genes of subfamily I (*Mal d 1.01* and -*1.02*) showed the highest expression level, which were 10 to 10,000 times higher than any other *Mal d 1* gene, and the combined total amount of RNA for these genes exceeded the amount of all other genes combined. *Mal d 1.01* was the most expressed gene, with a normalised expression of approximately 60 A.U. in the fruit peel of ‘Florina’. *Mal d 1.03C* was least expressed gene, with a transcript level close to the limit of detection.

The variations in gene expression between fruit tissues were evident, with the expression in the apple peel being significantly higher than in the flesh for both cultivars. The genes of subfamily V were the only exception, as *Mal d 1.11A* and -*1.11B* showed similar levels of expression in the peel and flesh, and *Mal d 1.12* was more expressed in the flesh than in the peel in ‘Gala’ fruits*.*

A comparison of the two cultivars revealed that ‘Florina’ generally shows a higher level of expression than ‘Gala’, which was consistent with a previous study on the expression of a limited number of *Mal d* genes [19]. Specifically, for 15 out of 20 genes, the expression was higher in ‘Florina’, with an average increase of 60%. The remaining 5 genes, *Mal d 1.03A*, -*1.03K*, -*1.06A*, -*1.06C* and -*1.07*, belonging to subfamilies III and IV, were more expressed in ‘Gala’ than in ‘Florina’.

## Discussion

The results obtained from the assay described in this paper provided insight into the role of individual isoallergens in Mal d 1-related apple allergy. The assay was based on a complete set of primers that were suitable for expression studies for each *Mal d 1* isoallergen gene using qRT-PCR. All primers passed several assessments of validation and optimisation and can now be used in a broad range of experiments. This approach can be relatively quickly adopted to other crops whose allergen genes have been mapped, a reference genome sequence is available and some knowledge of the genes and allelic diversities is available, such as for the peach *Pru p 1* genes [6,22,26].

### The qRT-PCR assay

The study of gene expression is an informative approach, and the first application of this assay confirmed *Mal d 1* as a heterogeneous family of genes that show distinct expression patterns despite having highly similar sequences. For single copy genes, the qRT-PCR technique is a highly sensitive and reproducible technique, with a large dynamic range compared with microarray approaches [23], without requiring a strong knowledge of bioinformatics, expensive equipment or particular expertise. qRT-PCR is also relatively cheap, particularly when SYBR green chemistry is used, as in this study, initial accurate primers are selected and an appropriate methodology is employed. Here we demonstrated the suitability of qRT-PCR for the assessment of a large gene family, whereby we obtained gene specificity through the use of sequence polymorphisms at the 3^′^end of at least one of the two primers and stringent PCR conditions.

The calibration method can seriously affect the results of qRT-PCR based assays. The “Standard curve method” accounts for variations in PCR efficiency among primer pairs [23], thus facilitating the comparison and integration of data from different primer pairs and experiments. The only disadvantage is the need to include a series of standard samples in each qRT-PCR experiment for all the tested genes, including reference gene, thereby slightly increasing cost and time compared with other methods.

### Clarifying the role of individual genes

Small changes in protein sequences might result in large differences in allergenic characteristics. A single amino acid change at a critical position in the epitope formation might completely alter the allergenicity of Mal d 1 proteins [27-29]. For Bet v 1, a difference of 7 amino acids between two isoallergens resulted in large differences in their allergenic and immunogenic properties: Bet v 1.0101 acted as a strong sensitiser, whereas Bet v 1.0401 was clearly associated with weaker IgE responses [30]. A similar situation likely exists in the apple, implying the existence of naturally hypoallergenic Mal d 1 isoallergens that induce no or mild IgE responses in allergic individuals. Currently, the allergenicity of single Mal d 1 isoallergens and their variants remains undetermined. Previous studies using gene expression approaches to obtain information on the differential expression within this gene family have characterised *Mal d 1.02* as the most expressed isoallergen gene [17-20]. However, our assay is a crucial step towards the identification of specific candidate genes for allergenicity within the *Mal d 1* family through an assessment of the presence and unequal dispersion of all the *Mal d 1* gene transcripts in apple fruits, thereby correlating differences in allergenicity among cultivars with differences in the expression of individual genes.

Indeed, the results obtained from the first application of this assay showed that 11 of the 31 *Mal d 1* genes were not expressed in ‘Florina’ and ‘Gala’ fruit. Therefore, the genes encoding these isoallergens are not involved in the allergenicity of ‘Florina’ and ‘Gala’ fruits and reasonably in the allergenicity of other cultivars, reducing the number of candidates for further assessments. This result is consistent with studies conducted in birch, where the *Bet v 1* genes from only two of 5 subfamilies were expressed in pollen [31].

Among the 20 isoallergen genes that were expressed, we observed a large variation in expression between the tissues, genes and cultivars. We detected a higher expression of *Mal d 1* genes in the peel than in the flesh. Therefore, it is reasonable to assume that peeling of apple fruits would remove most of the Mal d 1 proteins. Nevertheless, peeling was helpful to a small portion of the apple allergic population [32], indicating that we cannot *a priori* exclude isoallergen genes based solely on their levels of expression. Indeed, a small amount of a highly allergenic isoform might provoke allergy due to high immune reactivity, whereas large amounts of low-immune-reactive or non-immune-reactive isoallergens might provoke no allergic responses [14]. Thus, isoallergens, such as *Mal d 1.11B,* which are equally expressed in the peel and flesh, should receive full attention, despite being expressed approximately 300 times less than *Mal d 1.01*. However, for individuals for which peeling fruit is helpful, we can further limit the number of potentially involved genes to those that are only expressed in the peel.

Apple cultivars differ in allergenicity according to oral provocations and skin prick tests [2,3]. The current assay improved our understanding of these differences, facilitating the correlation of expression profiles across *Mal d 1* genes to allergy responses across cultivars. ‘Florina’ exhibited lower skin prick test responses than ‘Gala’ in prick-to-prick tests on whole fruits [24]. Most of the *Mal d 1* isoallergen genes were more expressed in the less allergenic cultivar ‘Florina’, suggesting that these genes play a minor role in allergy, whereas genes that are more expressed in ‘Gala’ could receive priority for further analysis (i.e., *Mal d 1.06A*, *- 1.06C*, *-1.03A*, *-1.03K* and *- 1.07*). The indication for *Mal 1.06A* is of special interest, as a possible role for this isoallergen has been previously suggested [8] based on a correlation between the allelic composition of *Mal d 1.06A* and the level of allergenicity of a small set of apple cultivars. Extending the application of our qRT-PCR assay to a broader set of cultivars with known allergenicities will provide further insight into this phenomenon. We are currently applying this approach to apple fruits in which the expression of a range of *Mal d 1* genes is supposedly silent [33,34] and for which the data from oral provocations are available (unpublished observations). Even if there is a general good correlation between proteins and transcripts abundance [35], quantitative information about protein content would be highly desirable. It is always difficult to analyse specific protein isoforms in the case of protein families, in particular for Mal d 1 because of their sensitive 3D structure [9]. Despite that, thanks to this work it will be possible to focus the protein studies only on the interesting proteins, trying to develop Mal d 1 isoallergens-specific IgE.

Immunotherapy can be effective for respiratory-based allergies, but studies have presented contradictory results for food allergies. The apple allergy cross-reactivity between pollen and foods has been exploited, but the effectiveness of pollen allergen immunotherapy on allergies to cross-reactive foods has not been confirmed [36]. Oral desensitisation treatment has also been examined for the first time, with promising results, using a mixture of fresh fruits from different cultivars [37]. Our assay might facilitate the further development of immunotherapies, as the allergy causing isoallergens could be identified at the individual level, thus leading to personalised proteins for use in immunotherapy. Beyond immunotherapy, our assay might also provide information for personalised diagnostics and recommendations on the safe consumption of specific cultivars.

### Regulation of gene expression and the biological roles of Mal d 1

The *Mal d 1* genes are grouped into subfamilies based on similarities in their genomic and amino acid sequences [6]. This grouping is consistent with similarities in the expression profiles of these genes. Similar expression profiles were observed: i) all the genes of subfamily II were not expressed in fruit; ii) the two genes of subfamily I were both highly expressed and iii) the genes of subfamily V were equally expressed in the peel and flesh, suggesting sequence similarities in the promoter regions of these genes.

The biological role of Mal d 1 proteins in plants remains unclear. These proteins are activated in response to many different abiotic and biotic stresses [38,39] and play a putative role in plant defence responses to pathogens [17,40,41]. Indeed, Mal d 1 proteins are also known as pathogenesis-related proteins of class 10 (PR-10s) [41]. However, the specific *Mal d 1* genes involved in stress responses have not been identified and whether these genes are the same genes involved in allergy remains unknown. Thus, it is critical to determine whether breeding for low allergenicity would have consequences in terms of plant susceptibility against pathogens or abiotic stresses and whether high resistance to stresses would have consequences in terms of allergenicity. In addition, other stresses associated with growth and storage conditions affect the Mal d 1 content in apples [2,3,42]. Therefore, exploiting our set of primers to study the variation of gene expression at different pre-/post-harvest conditions or after exposure to biotic/abiotic stresses might contribute to defining a “hypoallergenic protocol” for apple production to favour the reduction of symptoms in patients with apple allergies.

## Conclusions

To date, patients with apple allergies deal with these condition through avoidance. To facilitate the normal consumption of essential components of a healthy diet containing apples, it will be useful to produce ‘allergy-friendly’ fruit. To obtain this goal, knowledge of the identification of Mal d 1 isoallergens that cause allergy is needed. The qRT-PCR assay described in this work facilitates the examination of individual genes. The first application of this qRT-PCR assay showed that only a portion of the *Mal d 1* genes is expressed in fruit. Moreover, the expressed genes showed great variation in expression among different tissues and cultivars. The data indicate that the presence and amount of specific Mal d 1 isoallergens determines allergenicity rather than total Mal d 1 content. For genes of specific interest, this assay might be further developed for allele (variant)-specific primer pairs, which, in turn, will promote the breeding of hypo-allergenic apple genotypes and support specific recommendations for fruit consumption, thereby reducing the impact of fruit allergies in a patient’s life.

## Methods

### Retrieval of *Mal d 1* sequences and their alignment

For the development of gene-specific primer pairs, all available sequence information for *Mal d 1* was used at both the gene and allele levels. Coding DNA sequences (cds) were retrieved from the literature [6-8,17,19], the ‘Golden Delicious’ genome sequence [22,43] and a BLASTN search in the NCBI database [44] using keywords and *Mal d 1* sequences as inputs. The coding sequences of 31 *Mal d 1* genes from the genome sequence of ‘Golden Delicious’ [6] were aligned using the Lasergene v8.0, MegAlign package (DNASTAR, Madison, WI, USA) and manually adjusted where necessary. A phenetic tree was produced using the same software, with default parameters and a neighbour-joining cluster algorithm (NJ). Single Nucleotide Polymorphisms (SNPs) among the different *Mal d 1* sequences were identified. Subsequently, a complete alignment of all available *Mal d 1* sequences was generated, and gene- and allele-differentiating SNPs were identified.

### Gene-specific primer design and validation

Gene-specific primer pairs were designed using the software Primer3 [45]; the targeted gene-differentiating SNPs were located at the 3^′^ end of at least one of the two primers (see Table 1) to ensure the primer’s ability to specifically amplify the target gene and, particularly, all known alleles of that target locus. Other criteria in the primer design included a primer length of 18–24 nucleotides, a guanine-cytosine content of 20-80% and a RT-PCR amplicon length of 80–200 base pairs. Each primer pair was tested for the formation of homo- and heterodimers using the software PrimerSelect of Lasergene v8.0 (default settings).

The gene specificity of each primer pair was validated in four ways. Firstly, for the *in silico* analysis, the primer sequences were blasted against the reference ‘Golden Delicious’ apple genome and the NCBI database. Secondly, to validate the primer specificity, the presence of a single PCR product after end-point PCR on genomic DNA was assessed. The PCR reactions were performed in a 17.5 μl volume containing 10 ng of DNA, 100 nM of gene-specific primers, 1X reaction buffer, 1.5 mM MgCl_2_, 100 μM dNTPs and 0.5 Unit AmpliTaq Gold® DNA Polymerase (Applied Biosystems, Foster City, CA, USA). The reaction included an initial 10 min denaturation step at 95°C, followed by 33 PCR cycles (45 sec at 60°C, 2 min at 72°C, and 30 sec at 95°C), with a final extension of 7 min at 72°C. The amplicons were visualised on an Image Station 440 CF (Kodak, Rochester, N.Y., USA) after electrophoresis on 1.5% (w/v) agarose gels and ethidium bromide staining. When non-specific bands were detected, the PCR conditions (primers concentration and annealing temperature) were optimised to obtain a single band. Thirdly, the primer specificity was validated through direct sequencing of all the amplicons from the gDNA of a set of 10 cultivars: ‘Florina’, ‘Gala’, ‘Santana’, ‘Elstar’, ‘Elise’, ‘Golden Delicious’, ‘Prima’, ‘Jonathan’, ‘Cox’ and ‘Ingrid Marie’. The sequencing reactions were performed at Bio-Fab Research, Pomezia, Italy and Greenomics, Wageningen, The Netherlands. The samples included tissues from low (‘Elise’ and ‘Santana’) and high (‘Golden Delicious’) allergenic apple cultivars [2,3,46], that have been used in other studies of apple allergenicity and in studies on the effects of genetic modification on the expression of allergen genes (‘Florina’ and ‘Gala’) [24,25]. Cultivars currently used in apple breeding (‘Cox’, ‘Elstar’, ‘Golden Delicious’, ‘Jonathan’ and ‘Prima’) were also included in these assessments. Subsequently, these sequences were analysed using Chromas Lite v.2.01, BLAST and MegAlign. Fourthly, the primer specificity was validated through qRT-PCR, using the shape of the melting curve as a criterion. Due to variations in length and nucleotide composition, each unique product was expected to have a unique melting temperature (Tm) and, consequently, a unique melting (or dissociation) curve. If the primer pair produced a single amplicon, the plot of the first derivative of the melting curve would contain a single sharp peak.

### Plant material, RNA extraction and cDNA synthesis

The developed qRT-PCR assay was applied to the fruits of two apple cultivars (‘Florina’ and ‘Gala’). The fruits were collected at the Cadriano Experimental Orchard of the University of Bologna (Italy) at the physiological ripening stage. For each genotype peel and flesh from 5 apples were separately pooled, frozen in liquid nitrogen and stored at −80°C until RNA extraction. In particular, the peel was carefully separated from the flesh, leaving only a thin layer of flesh attached to the peel. Two different RNA extractions (biological replicates) were performed from each pool. The cDNA was synthesised according to previously published methods [19].

### Absolute quantification of the expression levels of the *Mal d 1* genes

Expression of *Mal d 1* genes was characterised through qRT-PCR using a StepOne Plus Real-Time PCR instrument (Applied Biosystems) with a SYBR green-based assay. Similar to all other intercalating dyes, SYBR green binds to any double-stranded DNA; therefore, the specificity of the primers was carefully assessed. Each reaction was performed in a total volume of 10 μl, containing 5 μl of Power SYBR® Green Master Mix 2X, 70–100 nM of each primer, 3 μl of a 1:9 dilution of the cDNA and PCR-grade water. The reactions were incubated at 50°C for 2 min and at 95°C for 5 min, followed by 40 cycles of 95°C for 15 sec and 60/63°C for 1 min, with data collection at each annealing step. The reactions were performed in triplicate (technical replicates). To ensure the specificity of the amplifications, each amplification reaction was followed by a melting phase, according to the default settings of the Step One Plus instrument (from 60°C to 95°C) and each melting curve was assessed for the presence of a single peak. The qRT-PCR assay included a standard curve for each target gene in the plate. Each standard curve comprised 6 serial 1:10 dilutions (in duplicate) of the amplicons obtained from a fixed amount of gDNA using gene-specific primers, starting from 100 ng. The curve correlated fluorescence signal (expressed as Ct values) to known amounts of amplicons in the 6 serial dilution samples and a regression line was plotted. Each assay also included a negative control performed in duplicate. The shape of the melting curves obtained from the standard curve dilutions (gDNA) and the samples (cDNA) indicates whether the amplified products are homogeneous and the melting temperature provides confirmation that the correct product has been specifically amplified.

The amplification efficiency (E) of each primer pair was estimated using the slope of the regression line, according to the equation: E=10^(−1/slope)^ - 1 [47]. The relative PCR efficiencies were evaluated for each target gene in relation to the reference gene to select an appropriate method for the analysis of the raw qRT-PCR data. To obtain the gene expression results, the raw data was transformed using the “Standard curve method” [23] and reported as relative expression levels; the transcript levels of the *Mal d 1* genes were normalised to the transcript levels of *actin* (primer sequences from [48]) and expressed as Arbitrary Units (A.U.). *Actin* was chosen after the comparison of its gene expression to other two putative reference genes: (1) *UBC* (UBiquitin-Conjugating enzyme, MDP0000223660), forward primer 5^′^-CGAATTTGTCCGAAGGCGT-3^′^; reverse primer: 5^′^-CAATGATTGTCACAGCAGCCA-3^′^, and (2) *GAPDH* (Glyceraldehyde 3-phosphate dehydrogenase, MDP0000835914), forward primer 5^′^-ATTGGCAGTGTGCGACGTT-3^′^ and reverse primer 5^′^-GGAGGAGTCAATGGTGGAGGA-3^′^). The comparison of the three putative reference genes was done in two biological replicates of peel and flesh of ‘Florina’ and ‘Gala’. The mean normalised expression levels and the standard error of the mean (SEM) were calculated using the two biological replicates. Univariate analyses of the differences between the mean values were performed using analysis of variance (ANOVA) with a 0.05 significance level.

## Authors’ contributions

GP and RP: participated equally in the design of the study, carried out the molecular genetic studies, participated in the sequence alignment, performed the statistical analysis and drafted the manuscript. PA and ST: participated in the design of the study and critically read the manuscript. GR: participated in the design and coordination of the work. MJMS: critically read the manuscript. WEW: participated in the design and coordination of the work and helped to draft the manuscript. All authors read and approved the final manuscript.

## Supplementary Material

Additional file 1 **List of *Mal d 1 *sequences included in the alignment.** Click here for file

Additional file 2 **Alignment of the 31 coding sequences of the *Mal d 1 *isoallergen genes.** Alignment of the 31 coding sequences of the *Mal d 1* isoallergen genes retrieved from the ‘Golden Delicious’ genome sequence. The alignment was performed using MegAlign (DNASTAR Lasergene v8.0). Each sequence was reported using the name of the related gene and the accession number from the Apple GBrowse - Malus x domestica v1.0 [22]. The mismatched residues in the consensus sequence are highlighted in white. Click here for file

Additional file 3 **Results of direct sequencing.** The results of the BLASTN analysis of the sequences retrieved from the direct sequencing of *Mal d 1* amplicons from 10 different apple cultivars. The table lists the targeted gene, the name of the primer pair and the ID of the most similar sequence in the database or in the Gbrowse of the apple genome for each of the cultivars [22]; the presence/absence of the SNPs in these reference sequences is also indicated. Click here for file
