# Erratum to: A qRT-PCR assay for the expression of all *Mal d 1* isoallergen genes

**DOI:** 10.1186/s12870-016-0757-9

**Published:** 2016-04-08

**Authors:** Giulia Pagliarani, Roberta Paris, Paul Arens, Stefano Tartarini, Giampaolo Ricci, Marinus J. M. Smulders, Eric W. Van De Weg

**Affiliations:** Wageningen UR Plant Breeding, Plant Research International, Droevendaalsesteeg 1, Wageningen, PB 6708 The Netherlands; Department of Fruit Tree and Woody Plant Sciences, University of Bologna, Viale Fanin 46, Bologna, 40127 Italy; Department of Paediatrics, University of Bologna, Via Massarenti 11, Bologna, 40138 Italy; Present address: Consiglio per la Ricerca e la sperimentazione in Agricoltura-Centro di Ricerca per le Colture Industriali, via di Corticella 133, Bologna, 40128 Italy

## Erratum

Unfortunately, the original version of this article [1] contained an error. The spelling of the author “Marinus J M Smulders” name was misspelt “Marinus MJ Smulders”.
