# Supplementary figures and images for: A qRT-PCR assay for the expression of all Mal d 1 isoallergen genes
Source: BMC Plant Biol. 2013 Mar 23;13:51. doi: 10.1186/1471-2229-13-51 (PMC3616815; doi:10.1186/1471-2229-13-51)

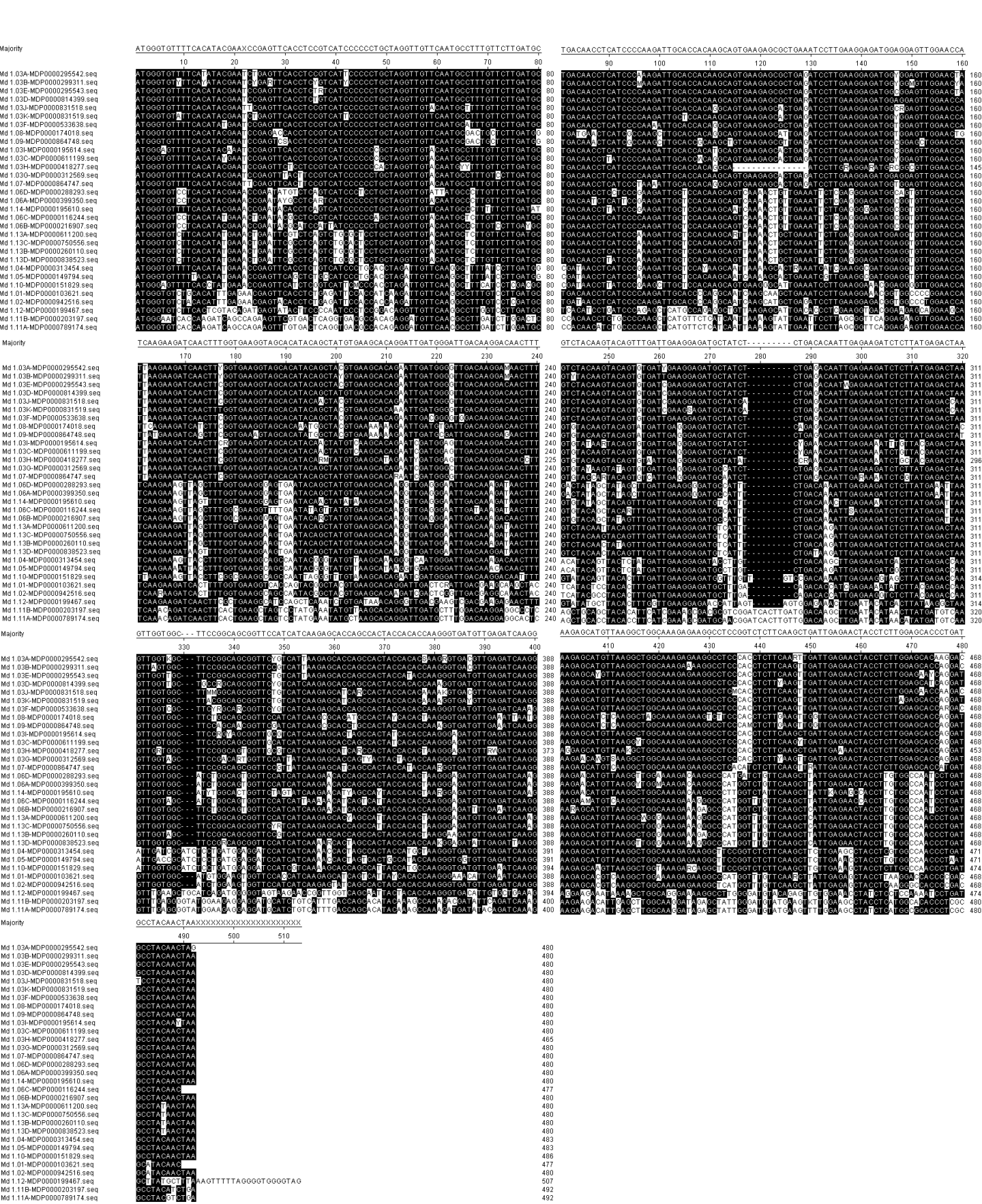

Supplement: Additional file 2 — Alignment of the 31 coding sequences of the Mal d 1 isoallergen genes. Alignment of the 31 coding sequences of the Mal d 1 isoallergen genes retrieved from the ‘Golden Delicious’ genome sequence. The alignment was performed using MegAlign (DNASTAR Lasergene v8.0). Each sequence was reported using the name of the related gene and the accession number from the Apple GBrowse - Malus x domestica v1.0 [22]. The mismatched residues in the consensus sequence are highlighted in white. [file 1471-2229-13-51-S2.pdf]
